# Interferon-γ-induced activation of Signal Transducer and Activator of Transcription 1 (STAT1) up-regulates the tumor suppressing microRNA-29 family in melanoma cells

**DOI:** 10.1186/1478-811X-10-41

**Published:** 2012-12-17

**Authors:** Martina J Schmitt, Demetra Philippidou, Susanne E Reinsbach, Christiane Margue, Anke Wienecke-Baldacchino, Dorothee Nashan, Iris Behrmann, Stephanie Kreis

**Affiliations:** 1 Signal Transduction Laboratory, University of Luxembourg, 162A Avenue de la Faïencerie, Luxembourg, L-1511, Luxembourg; 2 Life Sciences Research Unit, University of Luxembourg, 162A Avenue de la Faïencerie, Luxembourg, L-1511, Luxembourg; 3 Hautklinik, Klinikum Dortmund GmbH, Beurhausstraße 40, Dortmund, 44137, Germany

**Keywords:** IFN-γ, Jak/STAT pathway, STAT1, Signaling, Melanoma, miR-29, Tumor-suppressor

## Abstract

**Background:**

The type-II-cytokine IFN-γ is a pivotal player in innate immune responses but also assumes functions in controlling tumor cell growth by orchestrating cellular responses against neoplastic cells. The role of IFN-γ in melanoma is not fully understood: it is a well-known growth inhibitor of melanoma cells *in vitro*. On the other hand, IFN-γ may also facilitate melanoma progression. While interferon-regulated genes encoding proteins have been intensively studied since decades, the contribution of miRNAs to effects mediated by interferons is an emerging area of research.

We recently described a distinct and dynamic regulation of a whole panel of microRNAs (miRNAs) after IFN-γ-stimulation. The aim of this study was to analyze the transcriptional regulation of miR-29 family members in detail, identify potential interesting target genes and thus further elucidate a potential signaling pathway IFN-γ → Jak→ P-STAT1 → miR-29 → miR-29 target genes and its implication for melanoma growth.

**Results:**

Here we show that IFN-γ induces STAT1-dependently a profound up-regulation of the miR-29 primary cluster pri-29a~b-1 in melanoma cell lines. Furthermore, expression levels of pri-29a~b-1 and mature miR-29a and miR-29b were elevated while the pri-29b-2~c cluster was almost undetectable. We observed an inverse correlation between miR-29a/b expression and the proliferation rate of various melanoma cell lines. This finding could be corroborated in cells transfected with either miR-29 mimics or inhibitors. The IFN-γ-induced G1-arrest of melanoma cells involves down-regulation of CDK6, which we proved to be a direct target of miR-29 in these cells. Compared to nevi and normal skin, and metastatic melanoma samples, miR-29a and miR-29b levels were found strikingly elevated in certain patient samples derived from primary melanoma.

**Conclusions:**

Our findings reveal that the miR-29a/b1 cluster is to be included in the group of IFN- and STAT-regulated genes. The up-regulated miR-29 family members may act as effectors of cytokine signalling in melanoma and other cancer cells as well as in the immune system.

## Background

In the past decade, small non-coding microRNAs (miRNAs) have been identified as new and important players in post-transcriptional gene regulation and ever since, their expression patterns and cellular functions have been investigated in cancer and other diseases [1,2]. MiRNA biogenesis can be differentially regulated [3], but generally starts with the generation of a primary (pri-) miRNA transcript (several thousand nucleotides long), which is subsequently processed into a 70–80 nucleotide precursor form (pre-miRNA), which, following nuclear export, is then cleaved into the ~22 nucleotide mature miRNA. One strand of the mature duplex is incorporated in the RISC (RNA-induced silencing complex), where it recognizes, binds to and represses mRNA target sequences [1]. MiRNAs are involved in many fundamental cellular processes as they are estimated to control >50% of all protein-coding genes in mammals [4]. Consequently, they have been implicated in the regulation of processes that promote cancer growth or conversely, in processes that might prevent cancers from developing. For instance, a cancer cell can emerge following the over-expression of so-called “oncomirs” (such as the miR-17-92 family, miR-21, -155, etc.) which down-regulate tumor-suppressors that control cell proliferation. On the other hand, miRNAs that function as tumor-suppressors by targeting cellular oncoproteins (such as let-7 family members, miR-15a, -16, -29, etc.) are frequently down-regulated in cancer tissues [5]. Therapeutics opting to replace the diminished tumor-suppressor miRNAs are currently being investigated and seem promising, as miRNAs exhibit high stability as well as high specificity for their target mRNAs [5,6].

A disease where patients are in urgent need of more effective treatments is advanced melanoma, the most aggressive form of skin cancer. Metastatic melanoma exhibit a severe resistance to therapy leading to 5-year survival rates of below 5% [7]. Around 50% of patients exhibit V600E mutations in the cellular kinase BRAF [8]. Recently, the BRAF-inhibitor Zelboraf® has been approved for treatment of late-stage malignant melanoma patients with V600E mutations, increasing life expectancy by several months [9,10]. Nevertheless, except excision at early stages, no curative therapies exist. Routinely, therapies against melanoma include IFN-α as an adjuvant treatment. Interferons are cytokines and constitute a major part of the innate immune response, but they are also recognized for their anti-proliferative properties. We and others have shown that the type-II-cytokine IFN-γ mediates growth inhibition of cancer cells by activating the transcription factor STAT1 [11,12]. After IFN-γ stimulation, STAT1 forms homodimers, which bind to GAS (IFN-γ-activated sequences) elements in the promoter regions of target genes. Very recently, we have found several miRNAs to be dynamically regulated following stimulation with IFN-γ [13]. One of the first connections between cytokine-induced Jak/STAT signaling and miRNAs has been established by Löffler *et al.*, who showed that IL-6 increased the expression of oncogenic miR-21 via STAT3 activation in myeloma cells [14]. The signaling cascades involving IL-6 or IFN-α/β/STAT3/miR-21 and others have been confirmed in several types of cancer and diseases [15-17].

In the current study, we have focused on the biochemical analysis of individual miRNAs regulated by IFN-γ which we have recently identified in a detailed-time course microarray experiment [13], and further concentrated on the interesting miRNA family miR-29 with its three mature members, miR-29a, -29b and -29c. It is transcribed into two primary transcripts, pri-29a~b-1 and pri-29b-2~c, from chromosomes 7 and 1, respectively. MiR-29 family members target the expression of proteins such as methyltransferases, extracellular matrix proteins and transcription factors [18-20], which are potentially involved in triggering enhanced invasion, migration or proliferation of cells. They are silenced or down-regulated in many types of cancer and have consequently been assigned tumor-suppressing properties, although in some cases also oncogenic roles have been reported [21,22]. Here, we demonstrate a specific and profound IFN-γ-induced, STAT1-dependent up-regulation of miR-29a and -29b in melanoma cells and importantly, also increased expression in primary melanoma patient samples (but not in metastatic tumors) whereas the second cluster pri-29b-2~c was consistently undetectable. Moreover, we provide evidence for the tumor-suppressing properties of miR-29 family members: inhibition of melanoma cell proliferation could be mediated by miR-29a, which down-regulated *CDK6* (cyclin-dependent kinase 6), an important player in cell cycle G1/S transition. Our findings identify the pri-29a~b-1cluster as a novel IFN-γ-regulated gene and open up new connections between miRNAs, interferon signaling and malignant melanoma, which could lead to novel concepts for potential treatment options in the future.

## Results

**Figure 1:**
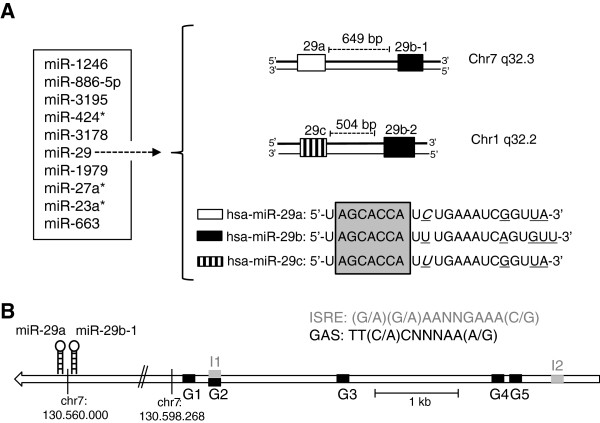
**Top 10 IFN-γ-up-regulated miRNAs.** (**A**) Ten miRNAs with highest positive fold changes (as determined by previous microarray experiments [13]) include miR-29 family members (left). Detailed time course profiles are shown in Additional file 1: Figure S1. The miR-29 family is transcribed from the respective antisense strand from two genetic clusters of chromosomes 7 (pri-29a~b-1) and 1 (pri-29b-2~c) (right). The three mature forms miR-29a/29b/29c share the same seed region (grey box). Differences between the mature sequences are underlined; a nucleotide difference between miR-29a and miR-29c is shown in italics. (**B**) The presumed pri-29a~b-1 promoter region [22-24] contains five GAS-elements G1-5 (TT(C/A)CNNNAA(A/G)) and two ISRE-elements I1-2 ((G/A)(G/A)AANNGAAA(C/G)) (GRCh37/hg19).

To investigate possible transcriptional regulations of miRNAs by STAT transcription factors, several melanoma cell lines were treated with IFN-γ for different time intervals and were subsequently analyzed by miRNA microarray as previously described [13]. The top 10 IFN-γ-induced miRNAs from a microarray experiment, which showed highest differential expression compared to untreated cells, and detailed time-course expression profiles thereof are depicted in Figure 1A and Additional file 1: Figure S1. For further analysis, we focused on the miR-29 family, as its mature members miR-29a and miR-29b showed the most robust regulations across all tested melanoma cell lines and because of its interesting properties regarding tumor biology. To identify the presence of potential IFN-response elements, we performed *in silico* screening of the promoter region 5 kb upstream of a putative transcription start of pri-29a~b-1 [22-24] and found five GAS-elements (TT(C/A)CNNNAA(A/G)) and two ISRE (interferon stimulated response element)-elements ((G/A)(G/A)AANNGAAA(C/G)) (Figure 1B). For control purposes, we selected miR-100, which was slightly down-regulated after IFN-γ stimulation and miR-25, whose levels were not induced in the microarray experiments. Other regulated candidates included several miRNA star sequences (“miR*” which here represents the miR strand, which arises from the 3’-arm of the hairpin, while the 5’-arm would be the guide or parent strand and is conventionally considered as “minor” product) which are currently being further assessed in our laboratory (Figure 1, Additional file 1: Figure S1).

### The pri-29a~b-1 cluster and mature miR-29a/29b are regulated by IFN-γ

**Figure 2:**
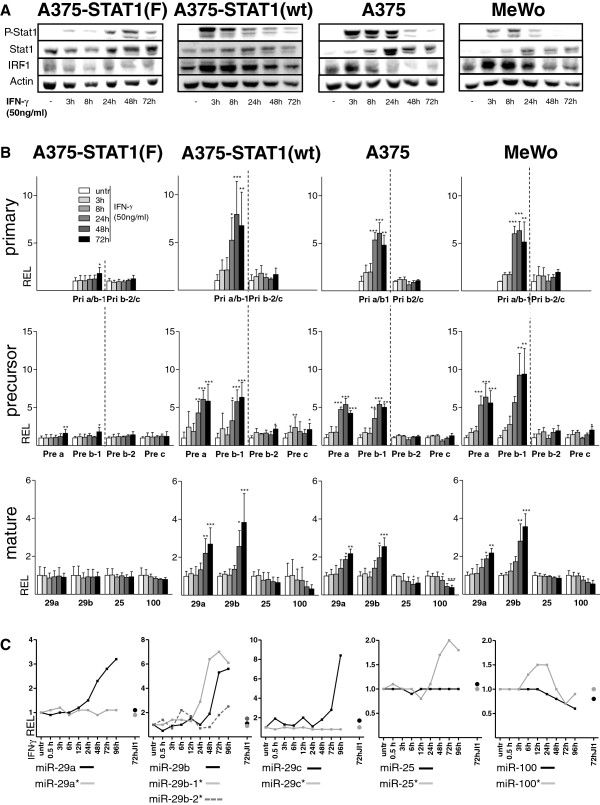
**Expression profiles of miR-29 clusters in melanoma cells.** A375-STAT1(F), A375-STAT1(wt), A375 and MeWo melanoma cells were stimulated with IFN-γ for different time points. (**A**) Western Blot analysis (representative blots of biological triplicates) confirms activation of P-STAT1 and induction of STAT1 and IRF-1 after IFN-γ stimulation while dominant negative A375-STAT1(F) cells show minor effects. (**B**) Time course study of miRNA-expression after IFNγ-stimulation. Graphs show relative expression (REL) from quantitative qRT-PCR data for the pri-29a~b-1 and the pri-29b-2~c clusters, the precursors pre-29a/29b-1/29b-2/29c and mature miR-29a/29b/25/100. Fold expression was calculated relative to the untreated control and SDs are shown for biological triplicates. Statistical significance was tested with one-way ANOVA, followed by a Dunnett Post-Hoc test with * p<0.05, ** p<0.01 and *** p<0.001. (**C**) MiRNA and miRNA* expression profiles in A375 cells derived from a more detailed IFN-γ time course miRNA microarray experiment including cells treated with JI1 (IFN-γ stimulation for 72h after pre-treatment with JI1, black and grey dots). Depicted are log2-values of the mean of duplicate microarray experiments.

For stimulation experiments with IFN-γ, melanoma cell lines MeWo and A375, as well as stably transfected A375 derivates were used. A375-STAT1(F) represent STAT1-dominant negative cells harboring a phenylalanine replacement of tyrosine residue 701 crucial for STAT1 phosphorylation and dimerization [12]. Thus, transcription of STAT1 target genes is abolished despite IFN-γ stimulation. The corresponding control cells A375-STAT1(wt) express the STAT1 wild-type construct instead [12]. To accurately assess the regulation of the miR-29 family by IFN-γ-induced STAT1, we performed time course experiments (Figure 2). Stimulation of A375, MeWo and A375-STAT1(wt) cell lines with 50 ng/ml of IFN-γ induced a prominent STAT1 phosphorylation, which decreased after 48h of IFN-γ treatment, whereas the STAT1-dominant negative cells A375-STAT1(F) only exhibited a delayed and weak P-STAT1 signal after IFN-γ stimulation (Figure 2A, see also [25]). Functional activity of the P-STAT1 transcription factor was confirmed by up-regulation of the STAT1 target genes IRF-1 and STAT1 itself, which showed induced expression after 3h and 8h, respectively.

Following stimulation, changes in miRNA expression levels were assessed by qRT-PCR (Figure 2B). A375, A375-STAT1(wt) and MeWo cell lines showed a strong and significant up-regulation (>5 fold) of the pri-29a~b-1 cluster, starting 24h after IFN-γ stimulation, while expression of the pri-29b-2~c cluster was not altered (Figure 2B, upper panel). Accordingly, miRNA precursors pre-29a and pre-29b-1 were also augmented whereas pre-29b-2 and pre-29c levels remained unaffected (Figure 2B, middle panel). Subsequently, significant up-regulation of both mature miR-29a and miR-29b following IFN-γ stimulation was confirmed (Figure 2B, lower panel). The two control amplifications of miR-100 (slightly down-regulated) and miR-25, which remained stable over time following IFN-γ stimulation confirmed the initial microarray-based expression profiles (Figure 2B, lower panel). Similar regulation patterns were also found in Jurkat and MT4 T-cells and in HEK293T kidney cells (for mature miR-29a, miR-29b, and miR-25, Additional file 2: Figure S2). Except for minor expression changes of the Pri/Pre-miR-29 species after 72h of IFN-γ treatment, no up-regulation was detected in the A375-STAT1(F) dominant negative control cells, clearly suggesting that STAT1 activity is required for the IFN-γ-induced regulation of miR-29 family members.

Figure 2C shows expression results of a detailed time course microarray experiment using IFN-γ-stimulated A375 cells. In parallel and as described before, cells had been pre-treated with Jak inhibitor 1 (JI1), which specifically inhibits Janus tyrosine kinases and subsequently prevented miR-29 up-regulation after IFN-γ stimulation [13].

Altogether, these data substantiate for the first time a time-dependent up-regulation of the expression of pri-29a~b-1 cluster as well as of the mature miRNAs miR-29a and -29b in melanoma cells, which is triggered by IFN-γ-induced STAT1 signaling.

### The miR-29b-2~c cluster is undetectable in melanoma cell lines, melanocytes and keratinocytes

**Figure 3:**
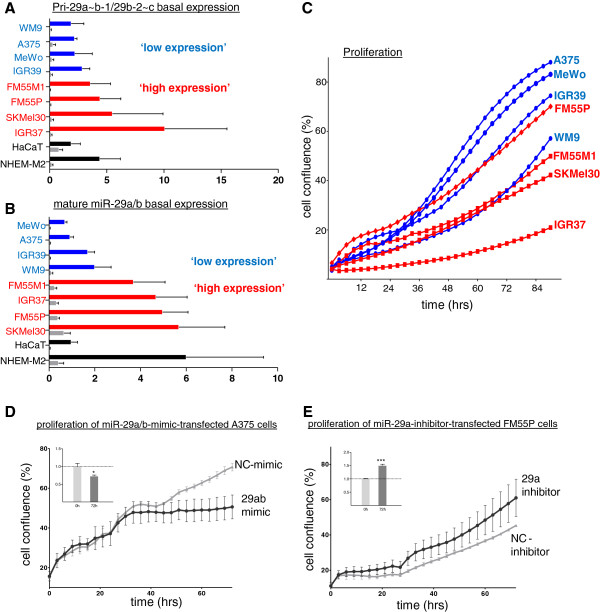
**Inverse correlation of miR-29a expression levels and melanoma proliferation.** Comparison of basal expression levels of (**A**) primary miRNA clusters pri-29a~b-1 (blue/red/black bars) and pri-29b-2~c (grey bars) and (**B**) mature miR-29a (blue/red/black bars) and miR-29b (grey bars) in NHEM-M2, eight melanoma cell lines and HaCaT keratinocytes. Graphs show 2^-Δct^ x10^2^ with SD of biological triplicates. (**C**) Mean growth curves of untreated melanoma cell lines over 4 days (biological quadruplicates). Melanoma cell lines with ‘low expression’ of pri-29a~b-1 and miR-29a show faster proliferation whereas cells with a relatively ‘high expression’ proliferate slower. (D,E) Proliferation assay of (black) mimic/inhibitor- and (grey) NC-mimic/NC-inhibitor-transfected cells over 72h in (**D**) A375 and (**E**) FM55P cells; representative graphs of four independent experiments. Error bars depict SDs of technical triplicates. The inserted graphs (upper left corners) show the mean confluence of 4 biological replicates at 0h and 72h time points of the proliferation assay. Depicted are ratios of confluence of 29ab-mimic/NC-mimic treated cells (D) and 29a-inhibitor/NC-inhibitor treated cells (E). Error bars show SEM. Significance was assessed by a two-tailed *t*-test with * p<0.05, ** p<0.01 and *** p<0.001.

As both miR-29 primary clusters as well as the mature miR-29a/29b showed different basal expression levels in stimulation experiments and are known to be differentially expressed in several types of cancer [26,27], we next analyzed the miR-29 basal expression profiles in a panel of melanoma cell lines, primary human melanocytes (NHEM-M2) and HaCaT keratinocytes (Figure 3A and B). Pri-29a~b-1 was strongly expressed whereas pri-29b-2~c was almost undetectable in all cell lines analyzed (Figure 3A). This is in accordance with previous studies reporting down-regulation of the pri-29b-2~c cluster in rhabdomyosarcoma [28] and B-cell lymphoma [23]. Also, mature miR-29a consistently showed higher basal expression levels than miR-29b in all cell lines examined (Figure 3B).

### MiR-29a/29b expression levels inversely correlate with growth behavior of melanoma cell lines

The classification of miR-29 as tumor-suppressor miRNA has been widely accepted and the possibility to use synthetic miR-29 as therapeutic agent in treatments of cancer seems to become increasingly realistic. Properties counteracting the development and spreading of cancer cells that have been observed *in vitro* and *in vivo* after miR-29 overexpression include reduced invasion and proliferation and induction of apoptosis [29,30]. These findings prompted us to analyze a potential correlation of basal miR-29 expression levels with cell growth. Proliferation of untreated melanoma cell lines was monitored over time (Figure 3C) in order to correlate the growth rate with miR-29a and pri-29a~b-1 basal expression levels obtained from cells harvested 96h after seeding. Melanoma cell lines were grouped in miR-29a and pri-29a~b-1 ‘low-expression’ (A375, MeWo, IGR39, WM9) and ‘high expression’ cell lines (FM55P, FM55M1, SK-Mel30, IGR37) (Figure 3A and B). Generally, cell lines with lower miR-29a showed an increased proliferation rate compared to lines with higher basal miR-29a levels (Figure 3C). Furthermore, the inverse correlation between pri-29a~b-1/miR-29a expression and the proliferation rate of melanoma cell lines might suggest a potential involvement of miR-29 in anti-proliferative effects on melanoma cells. To follow up these findings, we applied miR-29a/29b mimics to A375 cells, which exhibit a relatively low miR-29a/29b basal expression and, *vice versa*, we applied a miR-29a inhibitor to FM55P cells, which have a high basal miR-29a/29b expression (Figure 3B). Proliferation assays with mimics and inhibitors and the corresponding amounts of scrambled controls, NC (negative control)-mimic and NC-inhibitor, corroborated that miR-29 indeed inhibited growth of melanoma cells: transfection of miR-29a/29b mimics caused a remarkable reduction of proliferation as compared to NC-mimic-transfected A375 cells (Figure 3D). In turn, FM55P cells, in which miR-29a was inhibited, proliferated faster than NC-inhibitor-transfected control cells (Figure 3E).

### MiR-29a/29b down-regulate *CDK6*, but not *PI3K*

MiR-29 is predicted to regulate more than 1000 human genes (TargetScanHuman 6.1). We have used a combination of several algorithms (TargetScanHuman 6.1, Diana-microT v3.0, microRNA.org) to compile a list of potentially interesting genes, which carry predicted miR-29 target sites. After detailed expression analysis of potential candidates in melanoma cells and initial screening for their response to miR-29 mimic and inhibitor treatment (data not shown), we concentrated on the *PI3K* regulatory subunit (gene: *PI3KR1*; protein: PI3K/p85α) and *CDK6*, which play important roles in cell cycle control, cellular signaling and thus, proliferation. Both have already been confirmed as miR-29 targets in several cancers [31-34].

**Figure 4:**
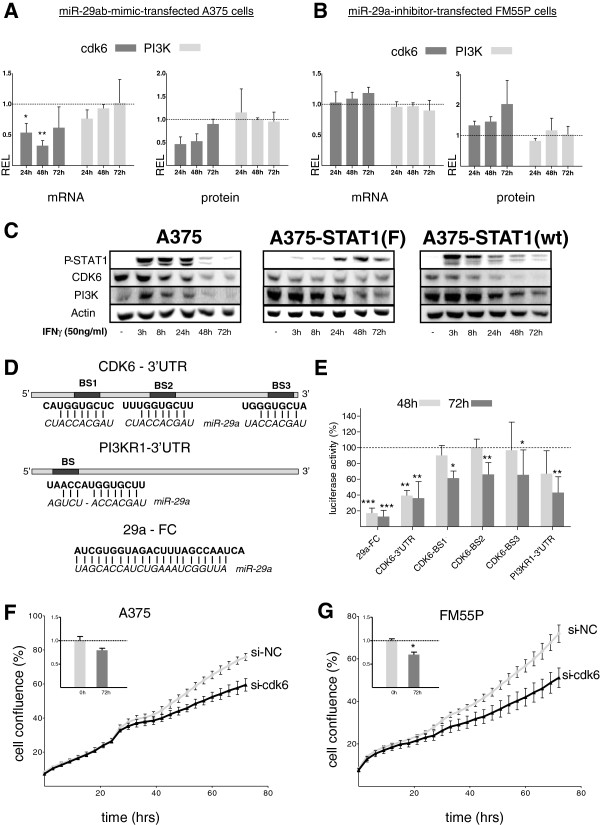
**Effects of miR-29 on target genes CDK6 and PI3K.** (**A**,**B**) relative mRNA and protein expression levels (REL) of miR-29 target genes CDK6 (dark grey) and PI3K (light grey), assessed 24h, 48h and 72h after mimic/inhibitor transfection compared to NC-mimic/NC-inhibitor controls; graphs show means of biological triplicates ± SD. (**C**) Down-regulation of miR-29 target proteins CDK6 and PI3K is observed after IFN-γ stimulation of melanoma cells. (**D**) Schematic overview of CDK6 and PI3KR1 luciferase constructs with positions of conserved miR-29a binding sites predicted by TargetScan (bold) in the CDK6-3'UTR (BS1-3) and PI3KR1-3'UTR (BS) and corresponding miR-29a sequences (italics). (**E**) Luciferase activity in A375 cells transfected with constructs containing the positive control miR-29a full complementary sequence (29a-FC), parts of CDK6- or PI3K1-3'UTRs or CDK6 single binding sites (BS1-BS3) and miR-29a mimic or NC for 48h and 72h. Relative luciferase activity of miR-29a-transfected samples was normalized to NC-mimic-transfected control samples (luciferase activity of NC-mimic transfected samples was set to 100%). Bars show means of biological triplicates ± SD for each construct. (**F**) A375 and (**G**) FM55P cells transfected with CDK6 siRNA (black) show reduced proliferation compared to cells transfected with siRNA NC (grey). Results were reproduced at least in biological duplicates. Inserted bar diagrams show the mean confluence of at least biological triplicates at 0h and 72h. Shown are confluence ratios of si-CDK6/si-NC ± SEM. Significance was assessed by one-way ANOVA followed by a Bonferroni Post-Hoc test (A,B,E) or by a two-tailed t-test (F,G). * p<0.05, ** p<0.01 and *** p<0.001.

To assess the effect of miR-29 on *CDK6* and *PI3K* expression in melanoma, mRNA and protein levels were examined after miR-29 mimic or inhibitor treatments by qRT-PCR and quantitative immunoblotting, respectively (Figure 4A and B). Combined transfection of miR-29a/29b reduced *CDK6* mRNA and protein levels in A375 cells as compared to scrambled controls whereas *PI3K* levels were not affected (Figure 4A). In agreement with that, knockdown of miR-29a in FM55P cells resulted in a slight up-regulation of *CDK6* levels while *PI3K* levels remained unchanged (Figure 4B). These data indicate that miR-29 is involved in down-regulation of *CDK6* protein while *PI3K* was not specifically targeted in melanoma cells. *CDK6* was also down-regulated in response to miR-29 induction after IFN-γ stimulation in A375 cells and A375-STAT1(wt) but not in A375-STAT1(F) cells, suggesting STAT1 dependency (Figure 4C). In contrast, PI3K levels were reduced in all three cell lines, hinting at STAT1-independent effects. To further prove regulation of *CDK6* by miR-29, we performed luciferase assays with reporter constructs containing part of the *CDK6* 3’-UTR, its three single miR-29 binding sites as predicted by TargetScan (http://www.targetscan.org), part of the *PI3KR1*-3’-UTR or the miR-29a full complementary sequence as a positive control (Figure 4D). Luciferase activity, as compared to the respective negative control, dropped by ~60 % for both time points in A375 melanoma cells when the *CDK6* 3’-UTR construct was co-transfected with miR-29a mimic. The corresponding single binding sites contributed to this suppression significantly with 38% (BS1), 34% (BS2) and 35% (BS3) (Figure 4E). This suggests that all three miR-29 binding sites partake in the suppression of *CDK6*. Surprisingly, the *PI3KR1* construct was also significantly suppressed by the miR-29a mimic in luciferase assays (Figure 4E) while only marginal effects had been observed on mRNA and protein level (Figure 4A,B). Taken together, these findings indicate that both *CDK6* and *PI3KR1* 3’-UTRs are directly targeted by miR-29 in melanoma cells; however, only *CDK6* suppression seems to be important in a cellular context. To further explore the relevance of reduced *CDK6* levels in the cell, we used siRNA against *CDK6* and assessed proliferation over 72h in A375 (Figure 4F) and FM55P cells (Figure 4G). Reduction of CDK6 mRNA and protein level (Additional file 3: Figure S3) led to a clearly diminished proliferation in both cell lines.

### MiR-29a and miR-29b are up-regulated in primary melanoma patient samples

**Figure 5:**
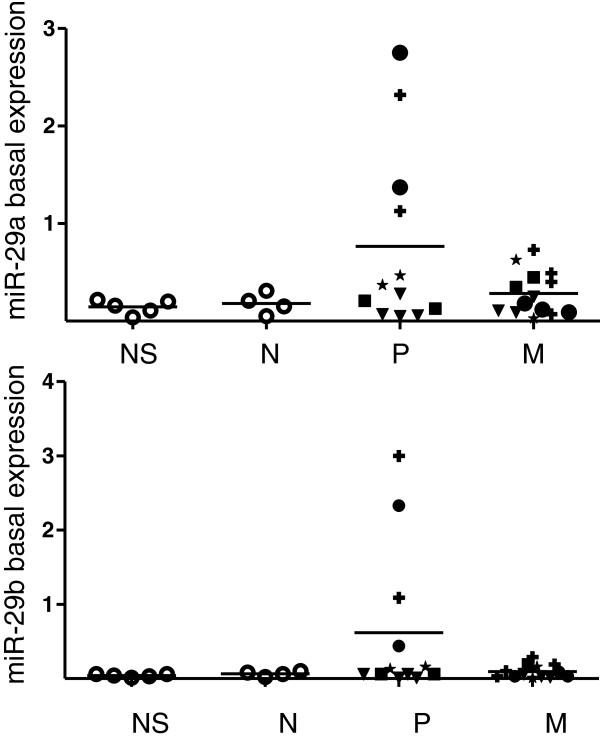
**miR-29 expression in patient samples.** Analysis of miR-29a (upper panel) and miR-29b (lower panel) basal expression of individual FFPE-patient samples from NS = normal skin, N = nevi, P = primary melanoma and M = metastatic melanoma. All graphs show 2^-Δct^ with Δct= (ct (miR-29a/29b)– ct (RNU5A)). Primary and metastatic tumor samples were sorted according to patients (P1-5: P1-circles; P2-rectangles; P3-crosses; P4-triangles; P5-asterisks).

Finally, we investigated miR-29a/29b expression profiles in FFPE melanoma patient samples from normal skin, nevi, primary and metastatic melanoma by qRT-PCR (Figure 5). Nevi represent the most appropriate control samples as they contain predominantly melanocytes while normal skin samples are mostly composed of keratinocytes. In comparison to healthy skin and nevi, both miR-29a and miR-29b showed an up-regulation in primary melanoma samples whereas in metastatic tumors, expression levels were only slightly enhanced compared to healthy controls. Closer sub-classification of the patient samples revealed, however, that only two of five patients demonstrated the enhanced miR-29a/29b expression, indicating that expression levels are heterogeneous and will have to be assessed in larger patient cohorts.

## Discussion

Generally, expression levels of miRNAs can be regulated transcriptionally, by epigenetic silencing or different turnover times [1,35,36]. The role of cytokines as inducers of miRNA expression has recently been proposed in several studies and examples for cytokine-induced miRNA up- or down-regulation include pro-inflammatory signaling molecules like TNF-α and IL1-β [37,38]. Interferons are central players in tumor-immune interactions [39,40]. In this context, the theory of ‘cancer immunosurveillance’, defined as the immunological protection of the host against development of cancer, has evoked much interest during the last decade: mediated by the host’s immune system, it is triggered by immune recognition of stress ligands or antigens expressed on transformed cells. IFN-γ has long been recognized for its crucial role in defense against viral and bacterial infections as well as in tumor control [40,41]. It primarily signals through the Jak/STAT pathway and activated STAT1 homodimers bind to GAS-elements in promoter regions of target genes, while IFN-α/β signal additionally through ISRE–elements. In our study, we have identified several GAS-elements in the proposed pri-29a~b-1 promoter region. IFN-γ stimulation of a control cell line expressing dominant-negative STAT1 (A375-STAT1(F)) did not cause an up-regulation of miR-29, providing strong evidence that STAT1 is indeed mediating IFN-γ-induced effects on miR-29 expression levels.

IFN-γ has anti-proliferative effects on cancer cells including melanoma [11,12] and we show here that miR-29a, which is induced by IFN-γ exhibited the same effects. Overall effects on growth were relatively small, but robust and reproducible, considering that we only manipulated levels of one miRNA and only used relatively small amounts of mimics/inhibitors (50 nM/150 nM) to be as close as possible to physiological relevance. However, IFN-γ may also facilitate melanoma progression: Zaidi *et al.* have shown that IFN-γ-producing macrophages are recruited to the UV-exposed skin and can stimulate proliferation and migration of melanocytes as well as induce expression of genes implicated in immunoevasion and survival. When added to transplanted melanoma, these skin-recruited macrophages enhanced the growth and survival of melanoma. All these effects were IFN-γ-dependent as demonstrated by antibody blocking of IFN-γ [42].

In our study, analysis of primary and metastatic melanoma patient samples revealed increased miR-29a/29b expression in some primary tumor samples in comparison to normal skin, nevi and metastatic tissue while all metastatic lesions had low levels of these miRNAs. Possibly, IFN-γ, which can be produced by macrophages, T cells and NK cells induces miR-29 expression via STAT1. miR-29a/29b were only up-regulated in two out of five primary melanoma patients. In this respect, it is interesting to note that IFN-γ producing macrophages have been observed in 70 % of melanoma samples [42]. A further evaluation of a larger panel of patient samples including early neoplasia and advanced metastatic stages is needed where a special focus will be placed on immune cell infiltration, interferon concentration and an interferon-responsive gene signature.

miR-29 has very recently been linked to interferon biology: it directly targets IFN-γ [43,44], the transcription factors Tbet and Eomes crucial for IFN-γ expression [19,44], and the receptor IFNAR1 [45], thereby drastically affecting immune regulation such as T cell polarization and thymic function.

While this manuscript was in preparation, IFN-γ involvement in the regulation of miR-29 expression was also reported by a group studying T cell activation and polarization in autoimmune diseases [44]. We here confirmed IFN-γ-induced miR-29 up-regulation in T cells (Jurkat and MT4, Additional file 2: Figure S2) and have also observed this effect in human embryonic kidney cells implying a regulatory mechanism of broader relevance. Interestingly, also type I interferons led to an up-regulation of miR-29 (Additional file 2: Figure S2).

Screening of a panel of melanoma cell lines for different miR-29 species and family members revealed that the pri-29b-2~c cluster was almost not expressed and that miR-29a exhibited a much higher basal expression level than miR-29b. In tumor cells, reduced miR-29 expression is frequently observed and diminished expression of miRNAs in general is often associated with enhanced oncogenesis [5,46]. The difference in pri-29a~b-1 and pri-29b-2~c expression levels, which we w?>have detected, is consistent with other types of cancer, in which the pri-29b-2~c cluster was mostly down-regulated [26-28]. The fact that miR-29 family members are often not expressed in cancer cells could be crucial for cancer control: miR-29 down-regulates important genes such as *CDC42*, *TCL-1* and *MCL-1*, which normally confer tumor-suppressing traits. In this context, anti-proliferating as well as anti-invasive and pro-apoptotic effects have been observed after miR-29 re-introduction in a variety of cancer cells [29,30]. In line with this, we show anti-proliferative effects of miR-29 and confirm for the first time *CDK6* as a direct miR-29 target in melanoma cells. This suggests that miR-29-mediated down-regulation of *CDK6* is involved in decreasing proliferation rates of miR-29a/b-mimic-transfected melanoma cells. SiRNA-mediated knockdown of *CDK6* resulted in reduced proliferation of melanoma cells similar to what has been shown for other cancer types [47,48]. *CDK6* plays a pivotal role in control of G1/S cell cycle transition [49] and loss thereof is a common event in neoplastic growth [50]. Noteworthy, *CDK6* has also been shown to be a direct miR-29 target in mantle cell lymphoma [31], acute myeloid leukemia [34] and cervical cancer [32].

The numerous anti-proliferative effects of IFN-γ in many cancers may in part be explained by a G1 arrest involving down-regulation of *G1/S cyclins* (cyclins A and E) and *CDK2/4*[12]. Accordingly, we find that IFN-γ as well as miR-29 exhibit anti-proliferative activities in melanoma cells involving down-regulation of cell cycle control players such as *CDK6*. The relevance of *CDK6* activity for melanoma growth is further emphasized by the fact that the tumor suppressor p16^INK4A^ (an inhibitor for CDK6 and 4) is deleted in about 50% of melanoma patients [51,52]. Here, we describe for the first time that *CDK6* is a direct target of miR-29 involved in regulating growth behavior of melanoma cells.

## Conclusion

**Figure 6:**
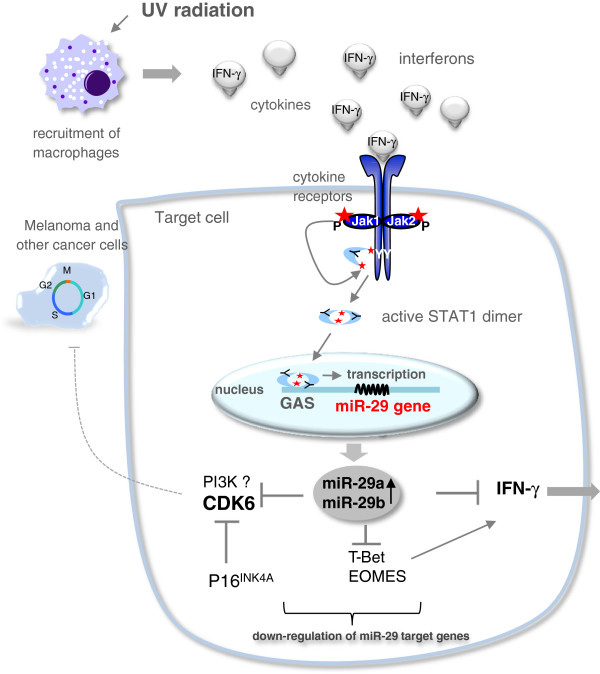
**The miR-29 family is involved in multiple cellular processes.** UV-radiation triggers the recruitment of macrophages to the skin, which secrete cytokines like interferon gamma (IFN-γ). By binding to its receptors, IFN-γ signals through the Jak/STAT pathway triggering subsequent activation of STAT1, which then binds to GAS-elements in the promoter region of target genes and initiates their transcription. IFN-γ-induced, STAT1-dependent up-regulation of miR-29 causes a down-regulation of CDK6, a novel miR-29 target gene in melanoma, which plays a crucial role in cell cycle G1/S-transition and thus growth control of cancer cells. The cell cycle inhibitor p16^INK4A^ is often deleted in melanoma and its function (inhibition of *CDK6*) might be compensated by miR-29a/b. IFN-γ-activating transcription factors T-bet and Eomes and IFN-γ itself are also targeted by miR-29.

Our study extends the current knowledge on the miRNA family miR-29, adding a novel regulatory loop of IFN-γ-mediated Jak/STAT signaling in melanoma cells. Figure 6 summarizes the proposed regulatory circuit involving IFN-γ and miR29: IFN-γ, which is e.g. secreted by macrophages following diverse assaults such as infections or UV light induces a STAT1-dependent up-regulation of miR-29, which in turn can down-regulate IFN-γ expression directly and indirectly via T-bet and Eomes. Down-regulation of cell cycle regulators like CDK6 contributes to IFN-γ-mediated growth arrest.

We report that the pri-29 b-2~c cluster is almost undetectable in melanoma, which might markedly reduce the ability of the miR-29 family to exhibit its tumor-suppressing properties in these cancer cells. The fact that miR-29a and miR-29b had enhanced expression levels in some primary melanoma patients but not in metastatic tumor samples is in line with many studies showing down-regulation or low levels of miR-29 in various advanced cancers [21,22,31,53]. We hypothesize that the reduced miR-29 expression in cancer cells could be a consequence of diminished IFN-γ signaling in those cells, which might already have escaped immune surveillance [41]. In regard to the proposed regulatory circuit, our study may open new connections between the immune system, miRNAs and growth control and thus, tumorigenesis.

## Methods

### Cell lines and patient samples

Melanoma cell lines A375 (American Type Culture Collection, ATCC), A375-STAT1(F) and A375-STAT1(wt) [25], FM55P and FM55M1 (European Searchable Tumor Line Database and Cell Bank, ESTDAB), IGR39 and IGR37 (Deutsche Sammlung von Mikroorganismen und Zellkulturen, DSMZ), MeWo (Dr. D. Schadendorf, Essen, Germany) and SK-Mel30 (Dr. M. Böhm, Münster, Germany) as well as the T cell lines Jurkat and MT4 (Dr. C. Devaux, Luxembourg) were maintained in RPMI 1640 supplemented with 10% FCS (PAA), 50 μg/ml penicillin, 100 μg/ml streptomycin and 0.5 mmol/l L-glutamine. The stably transfected A375 cell clones A375-STAT1(F) and A375-STAT1(wt) were grown under selective pressure with 400 μg/ml Geneticin (G418, Gibco). HaCaT keratinocytes (Dr. N. Fusenig, Heidelberg, Germany) and HEK293T were grown in DMEM supplemented with 10% FCS, 50 μg/ml penicillin, 100 μg/ml streptomycin and 2.5% HEPES. NHEM-M2 (normal epidermal human melanocytes, PromoCell) were cultured in melanocyte growth medium M2 (PromoCell) and harvested after reaching ~50% confluence in a 10 cm^2^ cell culture dish. All cells were maintained in a humidified atmosphere with 5% CO_2_ and were routinely tested to be mycoplasma-negative by PCR. Reagents and media were purchased from Lonza unless specified otherwise.

Ethical approval for use of the patient FFPE (formalin-fixed paraffin-embedded) and healthy control samples was obtained by the Ethical review board, Freiburg, Germany (Reference 196/09). Collection, histopathological analysis, fixation and RNA extraction were performed as described before [54]. In total, RNAs of 5 healthy skin samples, 4 benign nevi, 12 primary and 14 metastatic melanoma samples were analyzed by qRT-PCR. The primary and metastatic samples were collected from different parts of the body from a total number of 5 melanoma patients. Basal miR-expression levels were calculated as 2^-Δct^ with Δct= (ct (miR-29a/29b) – ct (RNU5A)) (Figure 5).

### IFN-γ stimulation, RNA extraction, and miRNA microarray analysis

For IFN-γ time course stimulation experiments, 100x10^3^ cells/well were seeded in 6-well plates (Greiner). Cells were either left untreated or stimulated with 50 ng/ml of IFN-γ (PeproTech) for the time periods indicated. 5 μM Jak inhibitor 1 (JI1, Calbiochem) pre-treatment was included (72h JI1-time point) in the detailed time course miRNA microarray experiment one hour before IFN-γ-stimulation. Samples for RNA extraction and protein lysates were collected altogether at the end of the treatment for further analyses by qRT-PCR and Western Blotting, respectively. Total RNA was extracted using TRIsure (Bioline USA, Inc.) and subsequently treated with DNaseI (New England Biolabs) as described before [54]. Quantity and purity of RNA samples were assessed using a NanoDrop ND-2000 spectrophotometer. Global miRNA expression levels were profiled on Affymetrix GeneCHip miRNA 2.0 Arrays as described before [13].

### Relative quantification of primary, precursor and mature miRNAs and mRNAs

For FFPE samples and cell lines, 250 ng of total RNA was reversely transcribed using the miScript Reverse Transcription kit (Qiagen) according to the supplied protocol. Real-time PCR was carried out on a CFX detection system (Bio-Rad). For quantification of mature miRNAs, 5 ng RNA input, 2x iQ SYBR Green Supermix (Bio-Rad) and 10x miRNA-specific primer assay (Qiagen) were used. To detect mRNAs, miRNA primary clusters and precursors, 2x iQ SYBR Supermix and 5 pmol gene-specific primers (for sequences see Additional file 4: Table S1) were used together with 50 ng (mRNA detection) or 125 ng (primary/precursor miRNAs) RNA input. PCR conditions for all qRT-PCR reactions were 95°C-3 min; 39x (95°C-15s; 60°C-30s); 95°C-1 min; 60°C-1 min, followed by a melt curve analysis (60°C to 95°C, increment 0.5°C for 20s) to confirm specificity of the PCR primers. If not stated otherwise, Ct-values for mRNA and miRNA species were normalized to at least three housekeeping genes: TBP (TATA binding protein), HPRT1 (Hypoxanthine phosphoribosyltransferase 1), CycloA (cyclophilin A) and β-Actin for mRNAs and primary/precursor miRNAs; RNU1A, RNU5A (RNA, U1A/5A small nuclear) and SCARNA17 (small Cajal body-specific RNA 17) for mature miRNAs. Based on the geometric mean of the three reference genes, a normalization factor was calculated for each sample using geNorm, a VBA applet for Microsoft Excel [55]. The relative amount of each target in each sample was then corrected by dividing its amount by the corresponding normalization factor. Fold changes were calculated by dividing the normalized relative amount of treated samples with the normalized relative amount of the untreated sample that served as a control. Statistical significance was tested with one-way ANOVA, followed by a Dunnett Post-Hoc test. Except for the FFPE patient samples, all experiments were performed at least in biological triplicates. P values of <0.05 (*), <0.01 (**) and <0.001 (***) were considered significant.

### Western blot analysis

Cell lysis, SDS-PAGE, ECL detection, stripping and re-probing was performed as previously described [56,57] using the following antibodies: Actin (C4, Milipore), Tubulin, IRF-1, STAT1 and CDK6 (Santa Cruz), P-STAT1 (Cell Signaling), p85α (PI3K) (Upstate) and the corresponding HRP-labeled (ECL detection, Cell Signaling Technology) or fluorophor-coupled (quantification, Li-cor Biosciences) secondary antibodies. For quantification of proteins, signal intensities were assessed with a Li-cor Odyssey Infrared Imaging System (Li-cor Biosciences) and analyzed with the provided software. CDK6 and p85α signals were normalized to the respective Tubulin loading controls.

### Real-time proliferation assays

25 x10^3^ cells/well of eight untreated melanoma cell lines were seeded in 12-well plates and harvested after 96h of real-time monitoring in the IncuCyte live-cell imaging system (Essen Bioscience), which photographed cells in phase contrast every 3h. RNA was extracted and miR-29 species were amplified by qRT-PCR as described before [54] and above.

### miRNA mimic/inhibitor transfection

100 x10^3^ cells/well were seeded in 6-well plates and transfected after 24h with 50 nM of each miR-29a and miR-29b mimics or with 150 nM miR-29a inhibitor or corresponding amounts of negative controls (Qiagen) using the DharmafectDuo transfection reagent (Dharmacon) according to the supplied protocol; efficient transfection was confirmed by qRT-PCR (Additional file 3: Figure S3). For miR-29 target gene expression, RNA and protein lysates were collected 24h, 48h and 72h after transfection and subsequently analyzed by RT-qPCR and western blot. Proliferation was monitored by the IncuCyte cell-imaging system as described above.

### CDK6 siRNA transfection

50x10^3^ cells were transfected with 75nM ON-TARGET siRNA or siRNA negative control (si-NC) 24h after seeding in 6-well plates using the HiPerfect transfection reagent according to the manufacturer’s instructions (Qiagen). Proliferation was monitored in the IncuCyte as described above. CDK6 mRNA and protein levels were assessed after 24h, 48h and 72h to confirm efficient down-regulation (Additional file 3: Figure S3C,D).

### Luciferase reporter gene assays

The parts of CDK6 and PI3KR1 (Phosphatidylinositol 3-kinase) 3’UTRs containing miR-29 binding sites, CDK6 miR-29a single binding sites and the miR-29a full complementary sequence were cloned into the pmirGLO Dual Luciferase miRNA target expression vector (Promega) downstream of the luciferase gene (see Additional file 4: Table S1 for primer sequences and oligonucleotides). A375 cells were seeded at a density of 50 x10^3^ cells/well in 24-well plates one day before transfection. Cells were transiently co-transfected with 500 ng plasmid and 50 nM miR-29a mimic or negative control for 48h and 72h. Samples were lysed with 1x Passive Lysis Buffer (Promega) and luciferase activity was measured using the Dual-Luciferase Reporter Assay System (Promega) according to the manufacturer’s instructions. Firefly was divided by Renilla activity and normalized to the negative control for each construct. Significance was assessed by one-way ANOVA followed by a Bonferroni Post-Hoc test with * p<0.05, ** p<0.01 and *** p<0.001.

## Competing interests

The authors declare that they have no competing interests.

## Authors’ contributions

The study was carried out in collaboration with all authors. MS, DP, SR and CM performed the laboratory experiments and analyzed the results. AWB performed the *in silico* analysis of the miR-29 promoter region and provided bioinformatic support. MS, DP and SR drafted the manuscript. DN provided the primary melanoma patient samples and scientific background about the disease. IB and SK developed the experimental design of the study, interpreted results and participated in writing and critical revision of the manuscript. All authors read and approved the manuscript.

## Supplementary Material

Additional file 1 **Figure S1.** Schmitt_et_al_2012_Contains a graphical representation of array results: Top 10 up-regulated miRNAs (as listed in Figure 1A) after IFN-γ stimulation for the indicated time periods and 72h JI1. Click here for file

Additional file 2 **Figure S2.** Schmitt_et_al_2012_Contains bar diagrams of qRT-PCR results: MiR-29a/29b up-regulation after IFN-γ-stimulation and unchanged miR-25 levels in A) HEK293T kidney and B) Jurkat T cells. C) MiR-29a/29b up-regulation after IFN-α-, IFN-β- and IFN-γ-stimulation (50 ng/ml) in MT4 T cells. Click here for file

Additional file 3 **Figure S3.** Schmitt_et_al_2012_Contains bar diagrams of qRT-PCR results an western blots: Tracking of miR-29a/29b mimics in A375 cells (A) and miR-29a suppression after inhibitor transfection in FM55P cells (B); and knock-down of CDK6 mRNA (C) and protein levels (D) in both cell lines. Click here for file

Additional file 4 **Table S1.** within Schmitt_et_al_2012_Contains primer sequences. Additional Figure legends: Schmitt_et_al_2012_ Contains additional Figure legends. Powerpoint documents. Click here for file
