# Supplementary figures and images for: Interferon-γ-induced activation of Signal Transducer and Activator of Transcription 1 (STAT1) up-regulates the tumor suppressing microRNA-29 family in melanoma cells
Source: Cell Commun Signal. 2012 Dec 17;10:41. doi: 10.1186/1478-811X-10-41 (PMC3541122; doi:10.1186/1478-811X-10-41)

## Slide 1
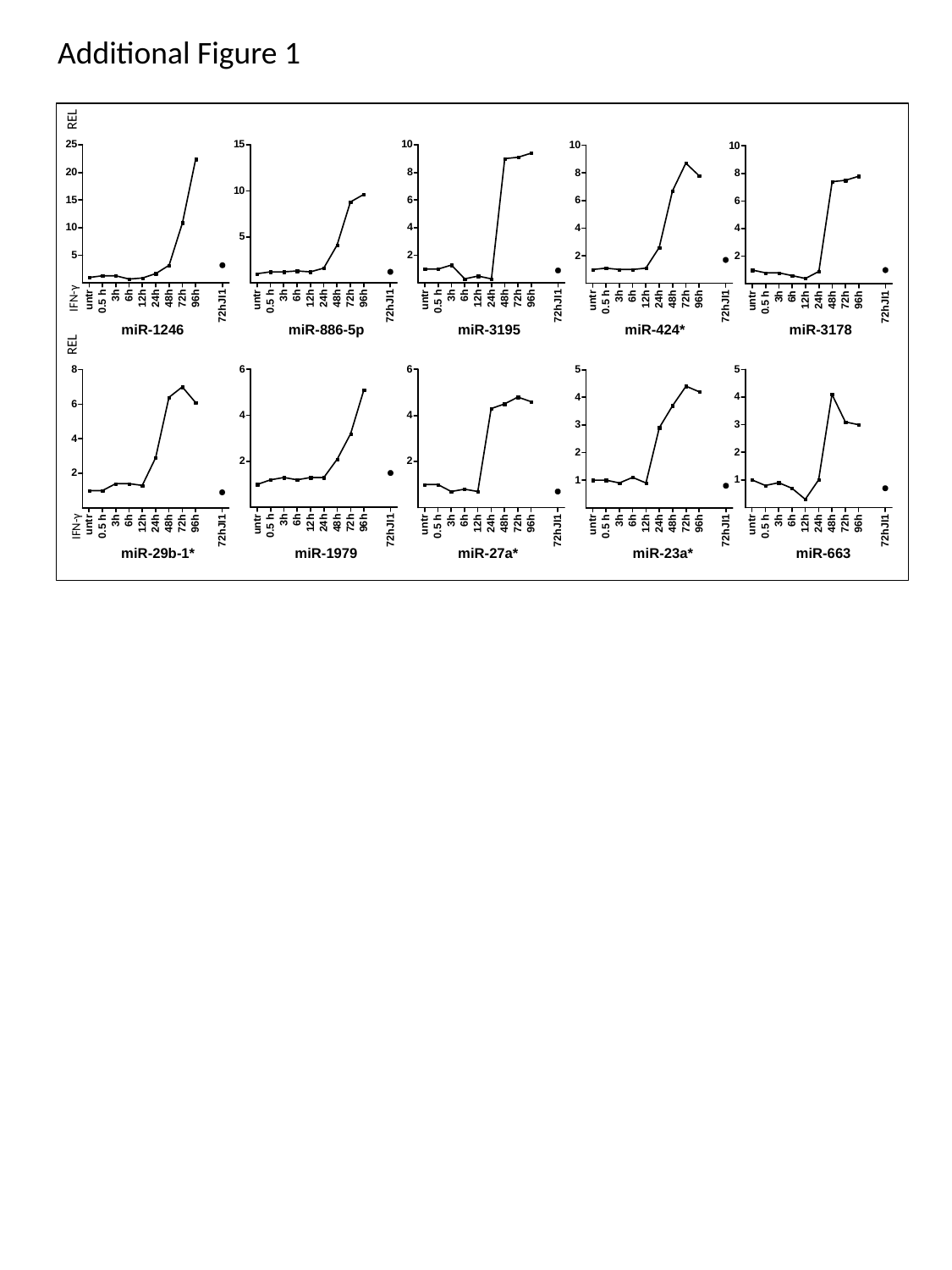

Additional Figure 1
REL
IFN-γ
miR-3195
miR-424*
miR-3178
miR-1246
miR-886-5p
REL
IFN-γ
miR-29b-1*
miR-1979
miR-27a*
miR-23a*
miR-663

Supplement: Additional file 1 — Figure S1. Schmitt_et_al_2012_Contains a graphical representation of array results: Top 10 up-regulated miRNAs (as listed in Figure 1A) after IFN-γ stimulation for the indicated time periods and 72h JI1. [file 1478-811X-10-41-S1.pptx]
