# Supplementary material for: Interferon-γ-induced activation of Signal Transducer and Activator of Transcription 1 (STAT1) up-regulates the tumor suppressing microRNA-29 family in melanoma cells
Source: Cell Commun Signal. 2012 Dec 17;10:41. doi: 10.1186/1478-811X-10-41 (PMC3541122; doi:10.1186/1478-811X-10-41)
Supplement: Additional file 2 — Figure S2. Schmitt_et_al_2012_Contains bar diagrams of qRT-PCR results: MiR-29a/29b up-regulation after IFN-γ-stimulation and unchanged miR-25 levels in A) HEK293T kidney and B) Jurkat T cells. C) MiR-29a/29b up-regulation after IFN-α-, IFN-β- and IFN-γ-stimulation (50 ng/ml) in MT4 T cells. [file 1478-811X-10-41-S2.pptx]

## Slide 1
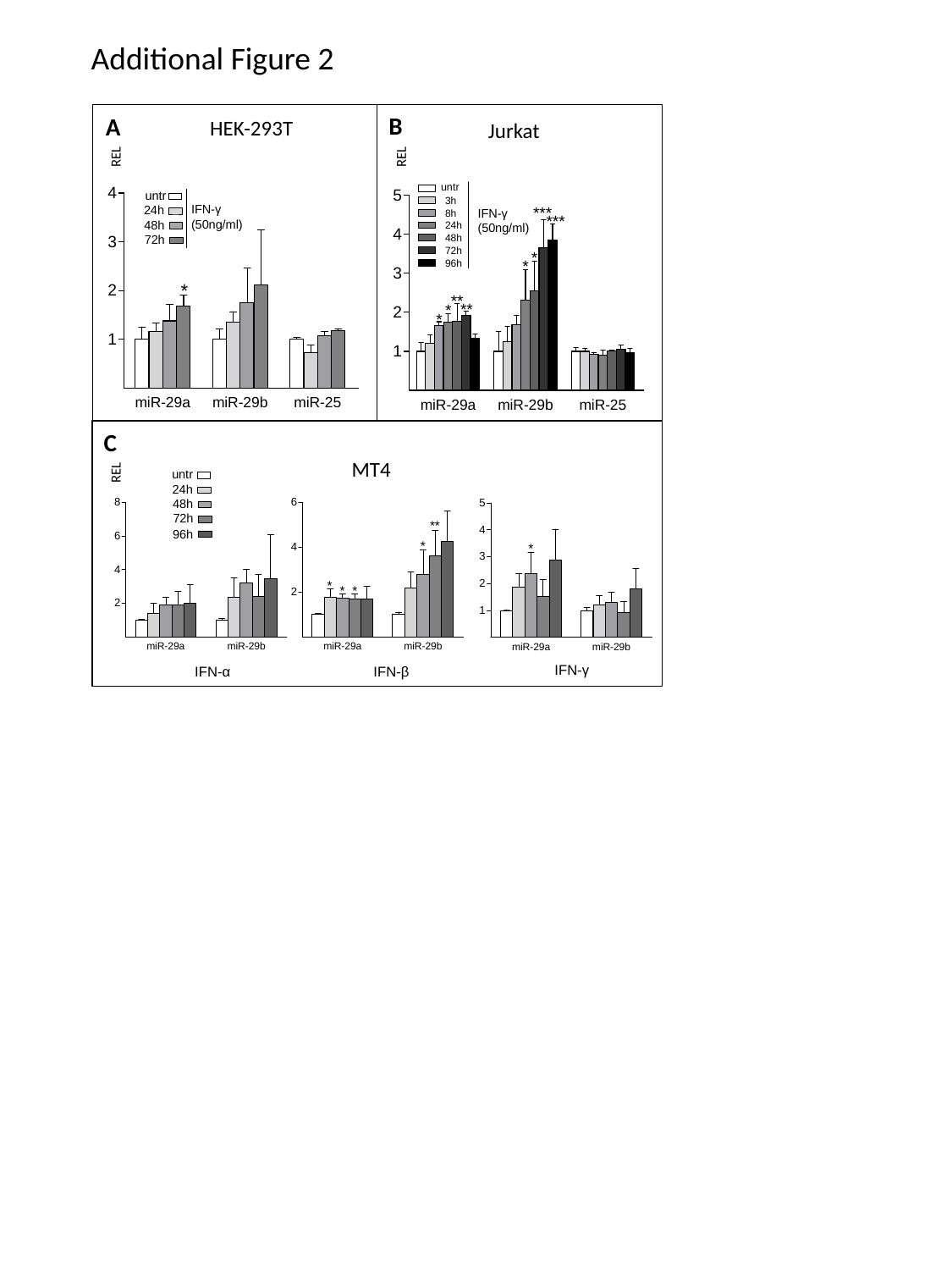

Additional Figure 2
B
A
HEK-293T
Jurkat
REL
REL
untr
untr
IFN-γ
(50ng/ml)
24h
IFN-γ
(50ng/ml)
48h
72h
C
MT4
untr
24h
48h
72h
96h
REL
IFN-γ
IFN-α
IFN-β
