# Supplementary material for: Interferon-γ-induced activation of Signal Transducer and Activator of Transcription 1 (STAT1) up-regulates the tumor suppressing microRNA-29 family in melanoma cells
Source: Cell Commun Signal. 2012 Dec 17;10:41. doi: 10.1186/1478-811X-10-41 (PMC3541122; doi:10.1186/1478-811X-10-41)
Supplement: Additional file 3 — Figure S3. Schmitt_et_al_2012_Contains bar diagrams of qRT-PCR results an western blots: Tracking of miR-29a/29b mimics in A375 cells (A) and miR-29a suppression after inhibitor transfection in FM55P cells (B); and knock-down of CDK6 mRNA (C) and protein levels (D) in both cell lines. [file 1478-811X-10-41-S3.pptx]

## Slide 1
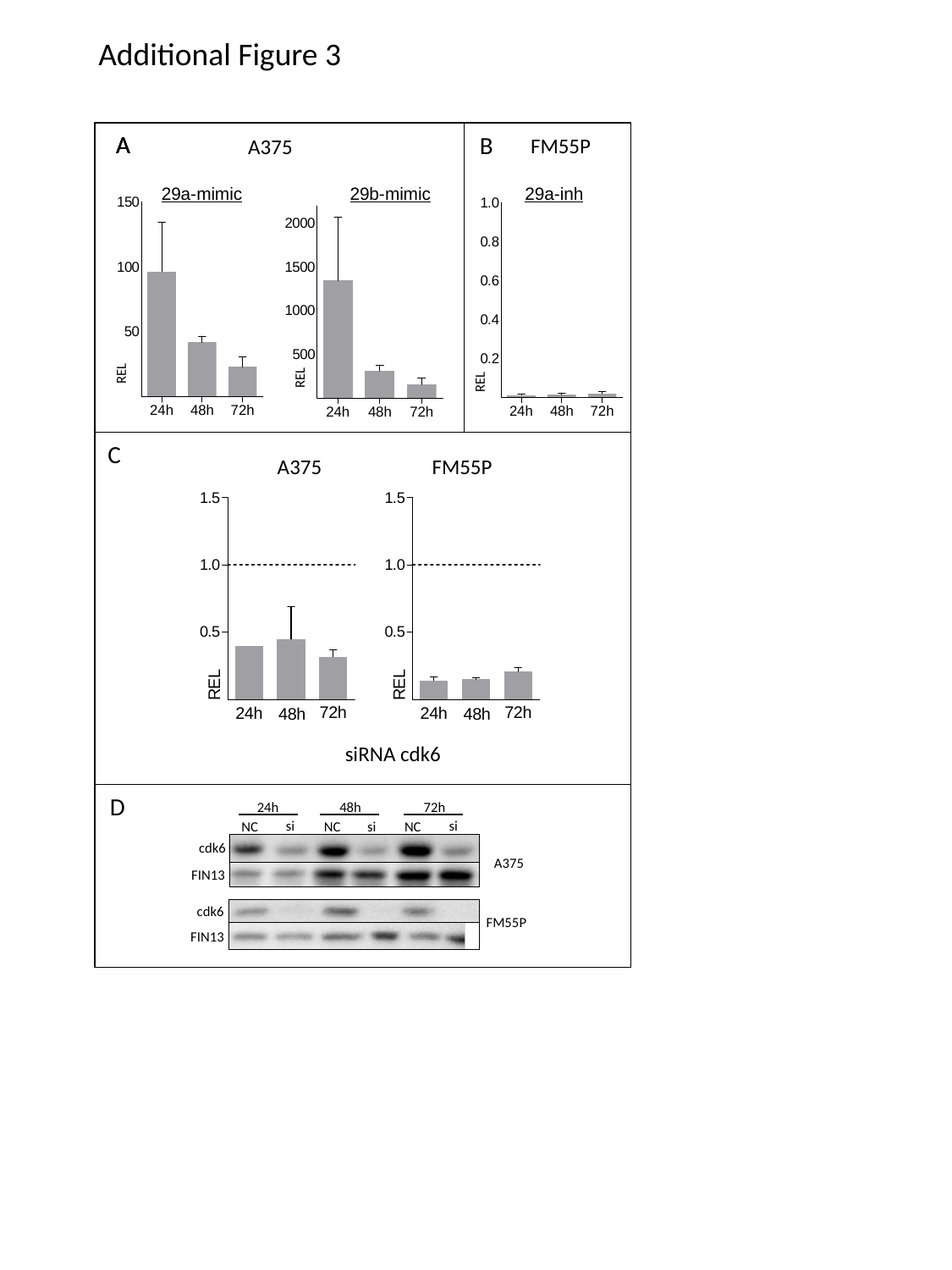

Additional Figure 3
A
A
A
B
FM55P
A375
29a-mimic
29b-mimic
29a-inh
REL
REL
REL
C
FM55P
A375
siRNA cdk6
D
24h
48h
72h
si
si
NC
NC
NC
si
cdk6
A375
cdk6
FM55P
FIN13
FIN13
