# Supplementary material for: Interferon-γ-induced activation of Signal Transducer and Activator of Transcription 1 (STAT1) up-regulates the tumor suppressing microRNA-29 family in melanoma cells
Source: Cell Commun Signal. 2012 Dec 17;10:41. doi: 10.1186/1478-811X-10-41 (PMC3541122; doi:10.1186/1478-811X-10-41)
Supplement: Additional file 4 — Table S1. within Schmitt_et_al_2012_Contains primer sequences. Additional Figure legends: Schmitt_et_al_2012_ Contains additional Figure legends. Powerpoint documents. [file 1478-811X-10-41-S4.docx]

**Additional Data**

**Additional Table S1**

**Additional Figure legends**

**Additional Figure S1**

**Additional Figure S2**

**Additional Figure S3**

**Additional References**

**Additional Table S1**

|  | amplicon |  | Sequence | amplicon  size (bp) |
| --- | --- | --- | --- | --- |
| primary  miRNAs | pri-29a~b-1 | F | 5’-GGGCTTTCTGGAACCAATCC-3’ | 68 |
|  |  | R | 5’-ACAATGCGATATCCTGTACAATTACAT-3’ |  |
|  | pri-29b-2~c | F | 5’-AAGAGCAAAATACACTCTTGAGTT-3’ | 63 |
|  |  | R | 5’-AACCCCCTTCTCTACTGTCAC-3’ |  |
| precursor  miRNAs | pre-29a | F | 5’-ATGACTGATTTCTTTTGGTGTTCA-3’ | 64 |
|  |  | R | 5’-ATAACCGATTTCAGATGGTGCTA-3’ |  |
|  | pre-29b-1 | F | 5’-CTTCAGGAAGCTGGTTTCATAT-3’ | 64 |
|  |  | R | 5’-TGATTTCAAATGGTGCTAGACA-3’ |  |
|  | pre-29b-2 | F | 5’-CTGGTTTCACATGGTGGCTTA-3’ | 62 |
|  |  | R | 5’-CACTGATTTCAAATGGTGCTAGATA-3’ |  |
|  | pre-29c | F | 5’-GGCTGACCGATTTCTCCTGG-3’ | 76 |
|  |  | R | 5’-TCCCCCTACATCATAACCGATTT-3’ |  |
| house  keeping  genes | HPRT1 | F | 5’-TGGACAGGACTGAACGTCTT-3’ | 77 |
|  |  | R | 5’-GAGCACACAGAGGGCTACAA-3’ |  |
|  | β-Actin | F | 5’-TGACCCAGATCATGTTTGAGA-3’ | 108 |
|  |  | R | 5’-AGTCCATCACGATGCCAGT-3’ |  |
|  | CycloA | F | 5’-CAGACAAGGTCCCAAAGACA-3’ | 139 |
|  |  | R | 5’-CCATTATGGCGTGTGAAGTC-3’ |  |
|  | TBP | F | 5’-ACCCAGCAGCATCACTGTT-3’ | 127 |
|  |  | R | 5’-CGCTGGAACTCGTCTCACTA-3’ |  |
| target  genes | CDK6 | F | 5’-CCAGCAGCGGACAAATAA-3’ | 92 |
|  |  | R | 5’-CCACAGCGTGACGACCA-3’ |  |
|  | PI3KR1 | F | 5’-ATACCCGCACATCCCAGG-3’ | 118 |
|  |  | R | 5’-TGTATTCTTTGCTGTACCGCTC-3’ |  |
| cloning primers for luciferase constructs | CDK6 3’UTR | F | 5’-TTTGCTAGCTGTATTAGTGTTTCTGCATTGCC-3’ | 1607 |
|  |  | R | 5’-TTTCTCGAGTTGGACAGTGATATTTCAACACC-3’ |  |
|  | PI3KR1 3’UTR | F | 5’-TTTGCTAGCACGTTCCTAAGCTGGAGTGCTT-3’ | 1552 |
|  |  | R | 5’-TTTCTCGAGCAGTCCAGAGCAGTGACAGTATGA-3’ |  |
| oligonucleotides for luciferase constructs | 29a FC | F | 5’-TCGACTAACCGATTTCAGATGGTGCTAT-3’ | - |
|  |  | R | 5’-CTAGATAGCACCATCTGAAATCGGTTAG-3’ |  |
|  | CDK6-BS1 | F | 5’-TCGACATGGAGAGCACCATGTGGACAAG-3’ | - |
|  |  | R | 5’-CTAGA CTTGTCCACATGGTGCTCTCCATG-3’ |  |
|  | CDK6-BS2 | F | 5’-TCGACACTCAAAGCACCAAAACAGAGCATTCTG-3’ | - |
|  |  | R | 5’-CTAGACAGAATGCTCTGTTTTGGTGCTTTGAGTG-3’ |  |
|  | CDK6-BS3 | F | 5’-TCGACTCATTCTAGCACCCAGTAAGACATCCAG-3’ | - |
|  |  | R | 5’-CTAGACTGGATGTCTTACTGGGTGCTAGAATGAG-3’ |  |

**Additional Table S1.** Primer sequences for qRT-PCR of primary and precursor miRNAs, housekeeping genes and mRNAs of target genes, cloning primers and oligonucleotides used for the construction of luciferase constructs. The precursor miRNAs pre-29a and pre-29b-1, which are both derived from the pri-29a~b-1 cluster were also induced after IFN-γ-stimulation, while pre-29b-2 and pre-29c levels remained unaffected (Fig. 2B). Nevertheless, precursor primers can also amplify primary clusters and up-regulation of miR-29 precursors may partially reflect the pri-29a~b-1 signal amplification. MiR-29c only bears one nucleotide difference to miR-29a (Fig. 1A) and is derived from the pri-29b-2~c cluster, which was almost undetectable in melanoma cells (Fig. 3B). Therefore, specific and correct qPCR amplification of miR-29c was not possible.

**Additional Figure legends**

**Additional Figure S1.** Top 10 up-regulated miRNAs (as listed in Fig. 1A) after IFN-γ stimulation for the indicated time periods and 72h JI1 (IFN-γ-stimulation for 72h after pre-treatment with JI1, time point marked by a black dot). Depicted are log2-values of the mean of duplicate Affymetrix miRNA microarray experiments as described before (12, in main document). Interestingly, miR-1246 showed the strongest up-regulation, however, we repeatedly failed to verify this regulation by qRT-PCR and northern blot analysis (data not shown). MiR-1246 has been reported to be highly expressed on different array platforms (Zhang et al. 2011; Piepoli et al. 2012), however, we assume that this could be due to unspecific hybridization to the miR-1246 probes or that current commercial primers are not suitable for qPCR amplification of this miRNA.

**Additional Figure S2.** Mature miR-29a/29b up-regulation after IFN-γ-stimulation and unchanged miR-25 levels in A) HEK293T kidney and B) Jurkat T cells. C) Mature miR-29a/29b up-regulation after IFN-α-, IFN-β- and IFN-γ-stimulation (50 ng/ml) in MT4 T cells. Graphs show relative expression (REL), all analyses were carried out as for stimulations of melanoma cells.

**Additional Figure S3.** Tracking experiments. To confirm efficient transfection, miR-29a/29b mimics were tracked in A375 cells (A) and miR-29a suppression after inhibitor transfection was tracked in FM55P cells (B) by qRT-PCR. Expression levels of miR-29a/29b were assessed 24h, 48h and 72h after mimic/inhibitor transfection; bars show means of biological triplicates with SD, relative to negative control (NC)-mimic/NC-inhibitor controls. Note that miR-29b transfection and/or amplification was more efficient than for miR-29a. Application of miR-29b inhibitor was not possible as the inhibitor itself was amplified by miR-29b primers in qRT-PCR, thus inhibition of miR-29b could not be properly monitored and controlled. However, as shown in Fig. 3B, miR-29b was generally expressed much lower than miR-29a, so that inhibition of miR-29a was more important concerning cellular effects. Furthermore, our initial experiments on combinatorial miR-29a/29b inhibition revealed no additional or synergistic effects on target genes compared to miR-29a inhibition alone (data not shown). To confirm efficient knock-down by cdk6-siRNA, cdk6 expression was tracked in A375 and FM55P cells by (C) qRT-PCR and (D) western blot. (C) Expression levels of cdk6 were assessed 24h, 48h and 72h after siRNA transfection; bars show means of biological triplicates with SD relative to negative control. (D) western blot for cdk6 confirms efficient knockdown of cdk6 in A375 (upper panel) and FM55P (lower panel) cells; si = siRNA cdk6; NC = negative control. Anti-FIN13 antibody: 1:3000 (Becton Dickinson).

**Additional References**

Piepoli, A., Tavano, F., Copetti, M., Mazza, T., Palumbo, O., Panza, A., di Mola, F.F., Pazienza, V., Mazzoccoli, G., Biscaglia, G. et al. 2012. Mirna expression profiles identify drivers in colorectal and pancreatic cancers. *PLoS One* **7**(3): e33663.

Zhang, Y., Liao, J.M., Zeng, S.X., and Lu, H. 2011. p53 downregulates Down syndrome-associated DYRK1A through miR-1246. *EMBO Rep* **12**(8): 811-817.
